# Ultrasound measurement of vastus lateralis and vastus medialis muscle parameters to identify chronic thyrotoxic myopathy

**DOI:** 10.1530/EC-23-0083

**Published:** 2023-10-05

**Authors:** Shi-en Fu, Rou-mei Wang, Xing-huan Liang, Jing Xian, Jie Pan, Xue-lan Chen, Cheng-cheng Qiu, Zhi-ping Tang, Ying-fen Qin, Hai-yan Yang, Li-li Huang, Ya-qi Kuang, Yan Ma, Zuo-jie Luo

**Affiliations:** 1 Department of Endocrinology, The First Affiliated Hospital of Guangxi Medical University, Nanning, China; 2 Department of Ultrasonic Diagnosis, The First Affiliated Hospital of Guangxi Medical University, Nanning, China; 3 Department of Neurology, The First Affiliated Hospital of Guangxi Medical University, Nanning, China; 4 Department of Endocrinology, The Affiliated Hospital of Guilin Medical University, Guilin, China

**Keywords:** chronic thyrotoxic myopathy, hyperthyroidism, ultrasound, vastus lateralis, vastus medialis

## Abstract

**Introduction:**

Chronic thyrotoxic myopathy (CTM) is a common, easily neglected complication of hyperthyroidism. There are currently no standard diagnostic criteria for CTM, and the ultrasonic characteristics of CTM-affected skeletal muscle remain unclear. Herein, we aimed to evaluate hyperthyroid patients for CTM by ultrasound and identify ultrasonic muscle parameter cutoffs for CTM diagnosis.

**Materials and methods:**

Each participant underwent ultrasonography. The original (muscle thickness (MT), pennation angle (PA), and cross-sectional area (CSA)) and corrected (MT/height (HT), MT/body mass index (BMI), CSA/HT, and CSA/BMI) parameters of the vastus lateralis and vastus medialis (VM) were evaluated. The diagnostic effectiveness of ultrasound for predicting CTM was determined using receiver operating characteristic (ROC) curve analysis. Our study included 203 participants: 67 CTM patients (18 males, 49 females), 67 non-CTM patients (28 males, 39 females) and 69 healthy controls (20 males, 49 females).

**Results:**

The CTM group had lower muscular ultrasonic and anthropometric parameters, higher thyroid hormone and thyroid-stimulating hormone receptor antibody (TRAb) levels, and a longer duration of hyperthyroidism than the non-CTM group (*P* < 0.05). The VM-PA, VM-CSA, VM-CSA/HT, and VM-CSA/BMI were lower in females than in males (*P* < 0.05). Free thyroxine (FT4) and TRAb both showed significant negative correlations with VM-MT, VM-MT/HT, VM-CSA, and VM-CSA/HT (*P* < 0.05). VM-MT/BMI and VM-CSA/HT, respectively, best predicted male and female CTM (AUC = 0.84, 0.85; cutoff ≤ 0.07, < 4.01).

**Conclusion:**

Ultrasound measurement of muscular parameters, especially in the VM, is a valid and feasible way of diagnosing and characterizing possible CTM in hyperthyroidism.

## Introduction

Hyperthyroidism is described as thyrotoxicosis caused by inappropriately high synthesis and secretion of thyroid hormones (1). The etiology of hyperthyroidism is classified into 12 categories, such as Graves’ disease (GD), toxic multinodular goiter (TMNG), and toxic adenoma (TA) (2). Hyperthyroidism is most commonly caused by an autoimmune disorder called GD in which thyroid-stimulating hormone (TSH) receptor antibody (TRAb) stimulates thyroid follicular cells, leading to thyrotoxicosis (1, 3).

The clinical manifestation of hyperthyroidism can involve skeletal muscles. Chronic thyrotoxic myopathy (CTM) is characterized by progressive muscular weakness, wasting, and atrophy in the limbs (4). In people with hyperthyroidism, the prevalence of CTM is reported to be 82% (5). Proximal myopathy is the sole presenting symptom in some patients with hyperthyroidism (6). Patients with CTM usually have a slow and insidious onset of proximal muscle involvement. Weakness of distal, respiratory, or even bulbar muscles is relatively uncommon (7). CTM patients often present with difficulty in rising from a squatting position, climbing stairs, or lifting their shoulders (8). These effects of myopathy increase the risk of related complications and adverse events, such as falls, frailty, decreased exercise capacity, and impaired functionality and independence. Therefore, early recognition and intervention are necessary to retard the progress of muscle weakness and prevent deleterious incidents in patients with CTM. At present, the diagnosis of CTM is predominantly based on physical examination and symptoms. However, mild or subclinical symptoms of CTM are sometimes overlooked in physical examination.

The circumference of a muscle is an indirect indicator of its quality. For example, a narrow hip measurement reflects small gluteal muscles (9). A controlled clinical trial found that the arm, forearm, chest, waist, gluteus, thigh, and calf circumferences of thyrotoxic patients were thinner than those of healthy controls (10). Previous studies have demonstrated the validity and repeatability of circumference measurements for assessing muscle mass in hyperthyroidism. However, previous studies did not differentiate between hyperthyroid patients with and without CTM or clarify the characteristics of CTM patients in terms of circumference measurements.

To date, there are no clinical guidelines specifying how to diagnose CTM. The loss of muscle mass strength and balance is an important feature of CTM (11). Muscle strength can be evaluated by grip strength and chair stands (12). Quantitative features of skeletal muscle architecture, including fascicle length, muscle thickness (MT), pennation angle (PA), and cross-sectional area (CSA), are important indexes reflecting physical performance and clinical outcomes (13, 14). Currently, muscle quality can be assessed by CT, dual-energy x-ray absorptiometry (DXA), bioelectrical impedance analysis (BIA), ultrasound (US), and MRI (12, 15). Although the gold standards for muscle quantification are MRI and CT, these techniques are expensive and time-consuming (in the case of MRI) or involve radiation exposure (in the case of CT) (16). MRI is considered the gold standard for measuring muscle composition; however, it has disadvantages such as high equipment costs, long scanning times, and lack of portability (17). Although CT has been found to have a strong correlation and good agreement with MRI in measuring muscle quality, it is expensive, exposes patients to radiation, and requires interpretation by radiologists (18). Regarding the criteria for sarcopenia, BIA and DXA were recommended to measure muscle mass by the Asian Working Group for Sarcopenia (AWGS) 2019 (19). However, DXA measures total lean tissue mass rather than skeletal muscle mass, and a patient’s hydration status may influence the final outcome (20). Additionally, DXA has some disadvantages, such as dependence on hydration, the thickness of soft tissues, and unclear mathematical equations; these drawbacks can result in misleading measurements of body composition and limit the clinical application of DXA (21). Our previous study, referencing the criteria of the AWGS 2014, attempted to use grip strength and pace as potential indexes to predict and diagnose CTM. However, the appendicular skeletal muscle mass index (ASMI) in DXA is not completely applicable to the diagnosis of CTM in our experiments, according to the suggestions of AWGS 2014 (22). Although BIA can distinguish intracellular water from extracellular water (23), BIA also has some limitations that can influence its outcomes, such as the chemical composition of fat-free mass, previous physical exercise, and food or fluid intake, although this method is widely used to evaluate body composition (24). In addition, volume overload or the presence of a pacemaker or bilateral hip or knee replacement can cause BIA to malfunction (25). B-mode US, with advantages such as a lack of ionizing irradiation, low cost, portable equipment, adequate performance in all patients, and extensive availability in clinics and hospitals, has been recognized in recent years as a potential method for assessing muscle mass and quality (13, 21, 26). The advantages of musculoskeletal ultrasound are well known: it is a simple, low-cost, real-time, radiation-free technique using portable equipment (26). US was first used in 1968 to measure the cross-sectional area and volume of skeletal muscles. More than 600 studies and 27,500 subjects have supported the validity and reliability of US as a tool for quantifying the size of skeletal muscles (27).

To date, no study has investigated the characteristics of muscular US in CTM and assessed its value in diagnosing this condition. The aim of this study was to sonographically assess the parts of proximal muscles in patients with CTM and to investigate the associations between muscular US parameters to explore their usefulness in diagnosing and predicting CTM.

## Materials and methods

### Study participants

This study recruited patients with newly developed hyperthyroidism and healthy controls between January 2022 and October 2022. The etiology of hyperthyroidism in patients from the study group is Graves’ disease. A total of 203 participants who visited the outpatient clinic were assessed. Informed consent was obtained from all participants.

### Ethics

The authors ensure that research involving human subjects complies with the Declaration of Helsinki. Written informed consent was obtained from all participants. The study was approved by the Ethical Committee of The First Affiliated Hospital of Guangxi Medical University (2021(KY-E-334)).

### Inclusion criteria

Patients were included if they met the following criteria: (i) newly diagnosed hyperthyroidism in accordance with the 2022 edition for Guidelines for Diagnosis and Management of Hyperthyroidism and Other Cause of Thyrotoxicosis by the Chinese Society of Endocrinology (2) and(ii) symptoms of progressive muscular weakness, wasting, or atrophy in addition to the general symptoms of hyperthyroidism and in the absence of other reasons for neuromuscular diseases. If a patient had mild or ambiguous manifestations of CTM, electromyography data were collected to test for myogenic damage to assist in the diagnosis of CTM (28). Myasthenia gravis, thyrotoxic periodic paralysis, central nervous system disorders, and other reasons for progressive muscular weakness, wasting, and atrophy were excluded on the basis of neostigmine experiments, anti-AChR antibody titers, MRI, electrolyte assays, and other methods of examination of the nervous system.

### Exclusion criteria

The following candidates were excluded: (i) those with other endocrine diseases such as diabetes mellitus or gonadal, adrenal, or hypophyseal disorders; (ii) those with severe chronic or acute diseases such as heart failure, end-stage or acute kidney disease, or hepatic insufficiency; (iii) those with other autoimmune, rheumatic, neuromuscular, or articular disease; (iv) those who abused drugs or alcohol or were on a long-term glucocorticoid regimen; (v) those with a history of thyroidectomy; (vi) those who were pregnant at the time of the study; and (vii) those who could not cooperate with or stand up for US and anthropometric measurements.

### Laboratory measurements

Overnight fasting before venous blood was withdrawn. The collected blood sample was centrifuged at room temperature for 10 min at 956 ***g***. Serum thyroid hormone concentrations were assessed using the chemiluminescent immunoassay (TEGEN, TESMI i-200, Shanghai, China). For this assay, the limit of detection for free triiodothyronine (FT3) was 3.6–6.0 pmol/L, free thyroxine (FT4) was 7.86–14.41 pmol/L, TSH was 0.34–5.65 mIU/L and TRAb was 0–1.5 IU/L.

### Anthropometric parameters

Anthropometric measurements were collected from all participants. In the early morning after an overnight fast, the participants lay awake, relaxed, and lightly clothed on a bed. Each participant’s blood pressure was measured twice with a sphygmomanometer on the right arm. The resolution of the sphygmomanometer was 1 mmHg. After having their blood pressure measured, the participants stood on a stadiometer scale to have their weight and height measured. The resolutions of the stadiometer and weighing scale were 0.1 cm and 0.1 kg, respectively. The body mass index (BMI) was calculated as weight in kilograms divided by height in meters squared (29). A flexible tape calibrated in centimeters with millimeter gradations was used to measure waist, gluteal, bilateral mid-upper arm, and thigh circumference in a standing position. The waist circumference (WC) measurement was taken at the level of the midpoint between the lower border of the ribcage and the uppermost lateral border of the ilium at the end of normal expiration. The gluteal circumference (GC) measurement was taken horizontally around the hips at the level of the posteriormost point of the buttocks. The mid-thigh circumference (MTC) measurement was taken horizontally at the midpoint between the anterior superior iliac spine and the proximal edge of the patella (31).

### Muscle ultrasonography

**Figure 1:**
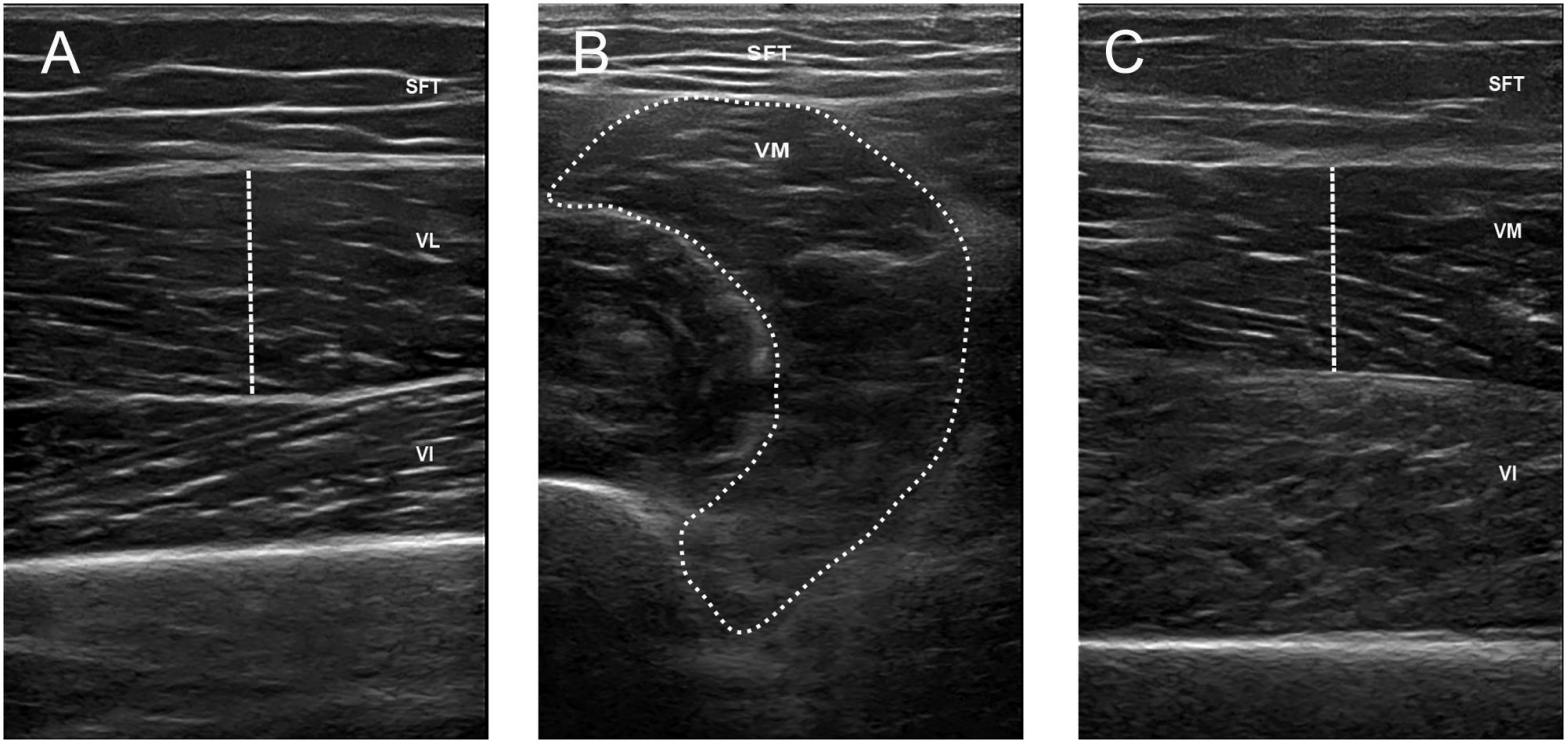
Examples of muscle ultrasound images in the HC group. A. Longitudinal section of the VL. B. Cross section of the VM. C. Longitudinal section of the VM. HC, healthy controls; SFT, subcutaneous fat thickness; VI, vastus intermedius; VL, vastus lateralis; VM, vastus medialis.

**Figure 2:**
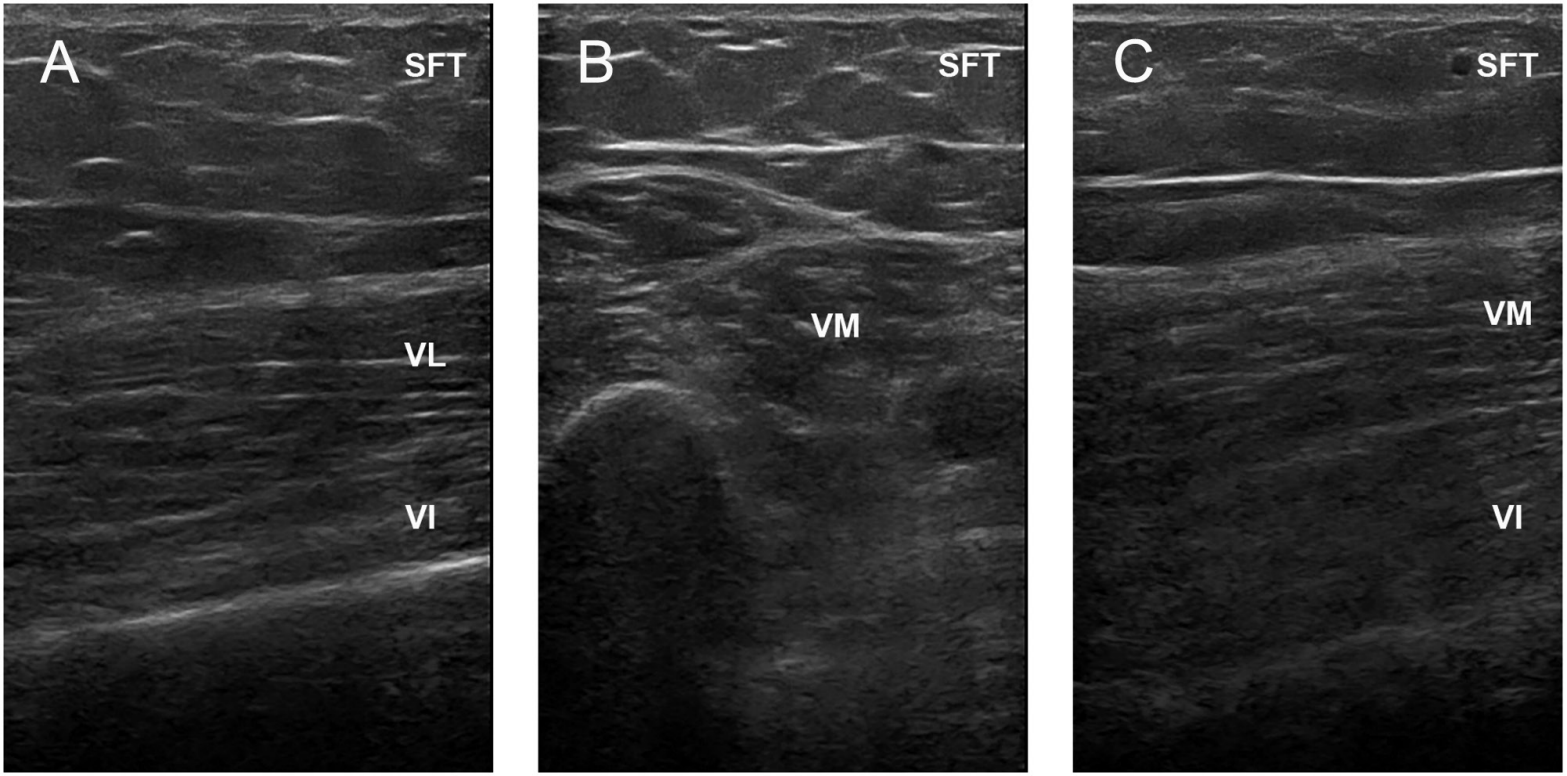
Examples of muscle ultrasound images in the CTM group. A. Longitudinal section of the VL. B. Cross section of the VM. C. Longitudinal section of the VM. CTM, chronic thyrotoxic myopathy; SFT, subcutaneous fat thickness; VI, vastus intermedius; VL, vastus lateralis; VM, vastus medialis.

**Figure 3:**
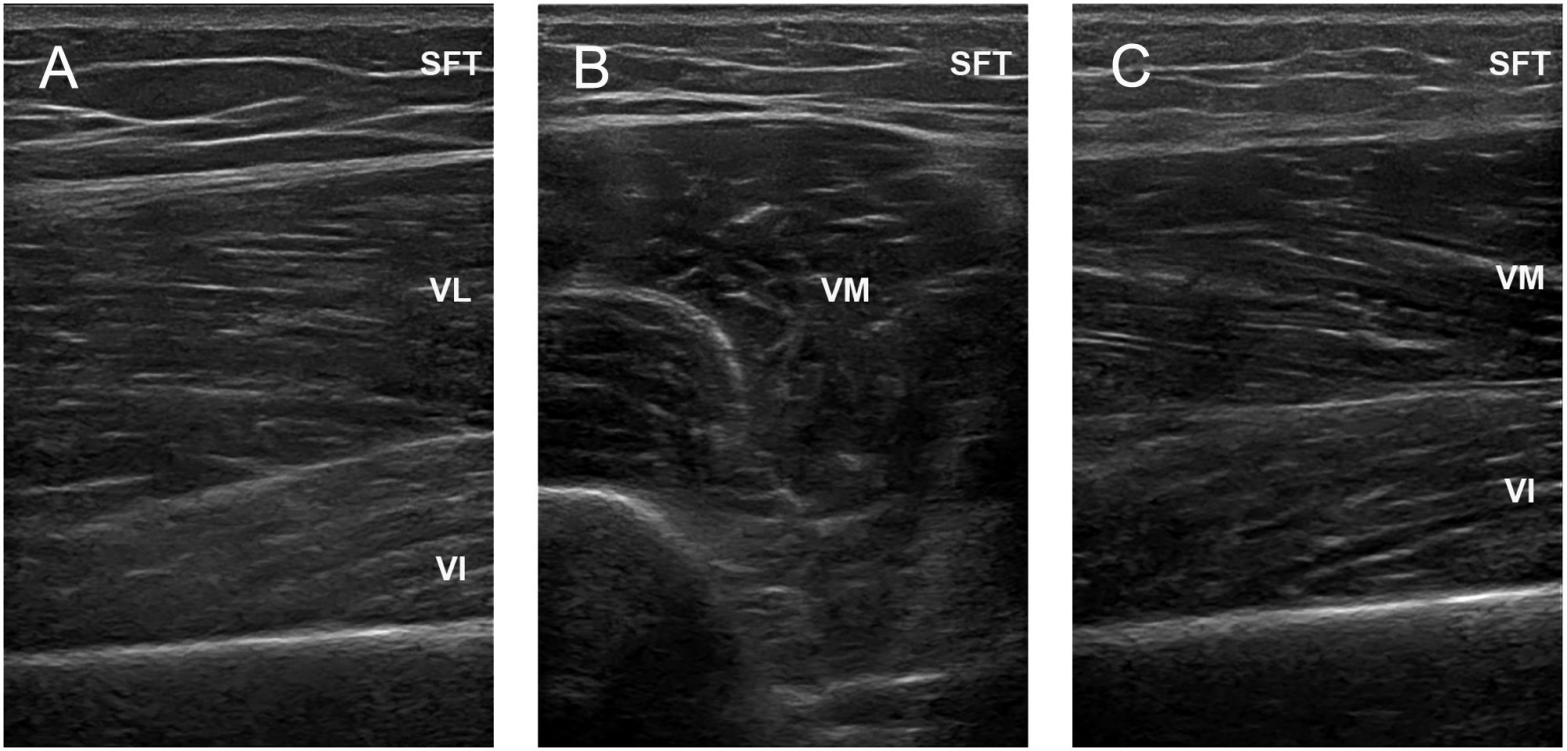
Examples of muscle ultrasound images in the non-CTM group. A. Longitudinal section of the VL. B. Cross section of the VM. C. Longitudinal section of the VM. CTM, chronic thyrotoxic myopathy; SFT, subcutaneous fat thickness; VI, vastus intermedius; VL, vastus lateralis; VM, vastus medialis.

US imaging was performed using a high-frequency linear probe with a muscle model (Clover 60, WISONIC), and images of the deltoid, iliopsoas, vastus lateralis, and vastus medialis muscle were taken separately with a transducer oriented in the longitudinal and transverse planes perpendicular to the skin. Standardized anatomical landmarks were also used to determine US measurement points in all participants. To ensure proper placement of the probe across repeated scans, each midpoint was clearly drawn on the skin with a marker pen. Participants lay in a supine position with their legs extended comfortably to assess the muscle quality of the proximal lower limbs. The vastus lateralis (VL) was imaged at 45% of the distance from the anterior superior iliac spine to the proximal end of the patella (Fig. 1A, 2A and 3A) (31). The vastus medialis (VM) was imaged at 65% of the distance from the anterior superior iliac spine to the proximal end of the patella (Fig. 1B, C, 2B, C, 3B and C) (31). Subcutaneous fat thickness (SFT) was measured as the distance between the dermis–adipose tissue interface and the muscle–adipose tissue interface (32). MT was defined as the distance between the deep and superficial aponeuroses in transverse images (33). PA was defined as the angle between the fibers and the axis at the insertion of the muscle fascicles into the deep and superficial aponeuroses (33). CSA was measured at the muscle–epimysium boundary around the muscle fibers (34). The dotted line represents where and how the authors measured MT and CSA for academic reproducibility in Fig. 1. In addition to measuring the raw values, we calculated the MT and CSA corrected by height (HT) and body mass index (BMI). The conditions were identical for all examinations. Each US parameter was measured 3 times by the same two specially trained sonographers, who were blinded to the study results and avoided measurement bias.

### Statistical analysis

The general information and clinical and US data of the three groups of subjects were compared using statistical analysis software SPSS (version 22.0). First, continuous variables were tested to determine whether they were normally distributed. Normally distributed continuous variables are presented as the mean ± s.d., while continuous variables with skewed distributions are expressed as the median and interquartile range. Categorical variables are recorded as frequencies. The two-sample *t*-test or analysis of variance (ANOVA) was used to compare continuous variables across two or three samples, respectively. If a significant difference was found between groups in ANOVA, the least significance difference test method was used for pairwise comparisons. For continuous variables with skewed distributions, the Wilcoxon rank-sum test or the Kruskal‒Wallis test with a Holm‒Bonferroni correction was used for comparisons across two or three samples, respectively, and the chi-square test or Fisher’s exact test was used for categorical variables. The correlations between general variables and clinical variables were determined using Spearman’s correlation analysis. A value of *P* < 0.05 was considered to indicate statistical significance. The utility of MT and CSA in diagnosing and predicting CTM was assessed by receiver operating characteristic curve analysis. The threshold with the maximum Youden index was considered the optimal cutoff value, and we calculated the sensitivity, specificity, positive predictive value, and negative predictive value (NPV) of this cutoff.

## Results

The study included 203 participants: 67 hyperthyroid patients with CTM (the CTM group; males:females = 18:49), 67 hyperthyroid patients without CTM (the non-CTM group; males:females = 28:39) and 69 healthy controls (HCs; males:females = 20:49). The mean ages were as follows: 37.39 ± 13.81 years for the CTM group, 34.79 ± 10.26 years for the non-CTM group, and 33.72 ± 10.91 years for the HC group. There were no significant differences in age, sex distribution, or HT. As expected, the CTM group had a longer duration of thyrotoxicosis and higher thyroid hormone and TRAb levels than the non-CTM group, as well as lower weight, BMI, WC, GC, and MTC (*P* < 0.05). The general characteristics of the study participants are shown in Table 1.

**Table 1:** Basic information and clinical data between the three groups of participants.

	CTM (*n* = 67)	Non-CTM (*n* = 67)	HC (*n* = 69)	K/F/*χ^2^*/Z	*P*
Age (years)	35.50 (27.75, 44.25)	35.00 (26.00, 40.00)	28.00 (24.00, 43.75)	2.78	0.249
Male, % (*n*)	18 (26.86)	28 (41.7)	20 (28.98)	3.99	0.136
Height (cm)	1.58 (1.55, 1.63)	1.60 (1.56, 1.68)	1.60 (1.55, 1.65)	4.69	0.096
Weight (kg)	50.35^ab^ (43.00, 55.00)	57.00 (49.00, 63.00)	56.50 (50.75, 63.25)	17.47	<0.001
BMI (kg/m^2^)	19.62^ab^ (17.84, 21.36)	21.76 (18.73, 23.62)	21.90 (20.00, 23.74)	18.67	<0.001
Duration (months)	3.23^a^ (1.40, 6.26)	1.20 (0.22, 2.76)	–	11.49	0.001
WC (cm)	74.00^b^ (69.00, 78.37)	75.14 (73.55, 76.93)	78.62 (73.00, 85.25)	8.93	0.012
HCirc (cm)	88.50^ab^ (83.50, 91.37)	90.50 (87.00, 94.50)	91.50 (88.00, 96.00)	16.92	<0.001
MAC (cm)	46.44 ± 5.51^ab^	49.43 ± 4.87^b^	51.24 ± 4.65	15.50	<0.001
FT3 (pmol/L)	24.95^ab^ (16.16, 38.66)	14.44^b^ (9.53, 21.30)	4.83 (4.27, 5.48)	49.48	<0.001
FT4 (pmol/L)	52.53^ab^ (35.90, 72.85)	32.94^b^ (22.60, 48.30)	11.10 (10.35, 12.56)	49.55	<0.001
TSH (mIU/L)	0.01^b^ (0.01, 0.01)	0.01^b^ (0.01, 0.01)	1.37 (1.03, 2.49)	91.26	<0.001
TRAb (IU/ L)	14.20^ab^ (9.78, 25.50)	5.75^b^ (2.42, 16.20)	0.25 (0.25, 0.25)	34.81	<0.001

^a^Compare with the group of non-CTM *P* < 0.05; ^b^compare with the group of health control *P* < 0.05.

BMI, body mass index; CTM, chronic thyrotoxic myopathy; FT3, free triiodothyronine; FT4, free thyroxine; HC, healthy control; HCirc, hip circumference; HT, height; MAC, mid-arm circumference; MTC, mid-thigh circumference; non-CTM, nonchronic thyrotoxic myopathy; TSH, thyroid-stimulating hormone; TRAb, thyroid-stimulating hormone receptor antibody; WC, waist circumference.

The CTM group had lower US-measured parameters than the non-CTM and HC groups. In the VL muscle, the MT, PA, MT/HT, and MT/BMI of the CTM group were lower than those of the non-CTM and HC groups, regardless of sex (*P* < 0.01). In the VM muscle, the MT, PA, CSA, MT/HT, MT/BMI, CSA/HT, and CSA/BMI of the CTM group were lower than those of the non-CTM and HC groups, regardless of sex (*P* < 0.01). Among male participants, the MT/BMI of the CTM group was lower than those of the non-CTM and HC groups (*P* < 0.05), but among female participants, there was no significant difference in MT/BMI between the CTM and non-CTM groups; the CTM group merely had a lower index than the HC group. Regarding sex differences in muscle US indexes, the data suggested that the VM-PA, VM-CSA, VM-CSA/HT, and VM-CSA/BMI in females were smaller than those in males (*P* < 0.05). For females, the average US-derived VM-PA, VM-CSA, VM-CSA/HT, and VM-CSA/BMI values were 15.03 ± 2.65°, 5.25 ± 1.20 cm^2^, 3.34 ± 0.73 cm^2^/m, and 0.26 cm^2^/kg/m^2^, respectively. For males, the corresponding values were 16.89 ± 2.15°, 7.30 ± 1.81 cm^2^, 4.38 ± 1.07 cm^2^/m, and 0.35 cm^2^/kg/m^2^, respectively. Tables 2, 3, and 4 show intergroup comparisons of the SFT, MT, PA, MT/HT, MT/BMI, CSA/HT, and CSA/BMI values.

**Table 2:** Ultrasound measurements of muscular parameters between the three groups of female participants.

	CTM (*n* = 49)	Non-CTM (*n* = 39)	HC (*n* = 49)	K/F	*P*
**VL**					
SFT (cm)	1.51 (1.22, 1.82)	1.50 (1.21, 1.86)	1.36 (1.13, 1.67)	2.46	0.292
MT (cm)	1.67 ± 0.35^ab^	1.92 ± 0.34^b^	2.20 ± 0.31	29.84	<0.001
PA (°)	15.70 ± 2.13^ab^	18.58 ± 1.73	19.87 ± 2.37	47.62	<0.001
MT/HT (cm/m)	1.07 ± 0.22^ab^	1.22 ± 0.22^b^	1.39 ± 0.20	27.86	<0.001
MT/BMI (cm/kg/m^2^)	0.08 ± 0.01^ab^	0.09 ± 0.01^b^	0.10 ± 0.01	13.19	<0.001
**VM**					
SFT (cm)	1.68 ± 0.48	1.72 ± 0.48	1.58 ± 0.52	0.93	0.396
MT (cm)	1.30 ± 0.33^ab^	1.50 ± 0.22^b^	1.72 ± 0.30	24.59	<0.001
PA (°)	15.35^ab^ (14.16, 16.59)	17.79^b^ (16.56, 18.96)	19.36 (17.87, 20.93)	55.27	<0.001
CSA (cm^2^)	5.48^ab^ (4.41, 6.03)	6.64^b^ (6.15, 7.76)	8.00 (7.42, 8.91)	76.48	<0.001
MT/HT (cm/m)	0.82^ab^ (0.72, 0.95)	0.94^b^ (0.81, 1.03)	1.07 (0.93, 1.23)	39.69	<0.001
MT/BMI (cm/kg/m^2^)	0.06^b^ (0.05, 0.07)	0.07 (0.06, 0.08)	0.08 (0.07, 0.09)	20.70	<0.001
CSA/HT (cm^2^/m)	3.45^ab^ (2.79, 3.82)	4.33^b^ (3.75, 4.89)	5.10 (4.67, 5.52)	76.98	<0.001
CSA/BMI (cm^2^/kg/m^2^)	0.26^ab^ (0.22, 0.31)	0.31^b^ (0.28, 0.39)	0.38 (0.33, 0.42)	49.09	<0.001

^a^Compare with the group of non-CTM *P* < 0.05;^ b^Compare with the group of health control *P* < 0.05.

BMI, body mass index; CSA, cross-sectional area; CTM, chronic thyrotoxic myopathy; HC, healthy control; HT, height; MT, muscle thickness; non-CTM, non-chronic thyrotoxic myopathy; PA, pennation; SFT, subcutaneous fat thickness; VL, vastus lateralis; VM, vastus medialis.

**Table 3:** Ultrasound measurements of muscular parameters between the three groups of male participants.

	CTM (*n* = 18)	Non-CTM (*n* = 28)	HC (*n* = 20)	K/F	*P*
**VL**					
SFT (cm)	0.93 ± 0.40	0.86 ± 0.32	0.82 ± 0.29	0.46	0.636
MT (cm)	1.79 ± 0.44^ab^	2.26 ± 0.49	2.35 ± 0.37	8.54	0.001
PA (°)	16.61^ab^ (14.80, 19.53)	20.02 (18.78, 21.51)	21.57 (20.61, 22.29)	28.34	<0.001
MT/HT (cm/m)	1.04^ab^ (0.89, 1.28)	1.29 (1.10, 1.54)	1.42 (1.23, 1.52)	12.45	0.002
MT/BMI (cm/kg/m^2^)	0.08^ab^ (0.07, 0.09)	0.10 (0.08, 0.11)	0.10 (0.09, 0.11)	13.99	0.001
**VM**					
SFT (cm)	1.09 ± 0.44	1.08 ± 0.38	1.01 ± 0.33	0.25	0.777
MT (cm)	1.36^ab^ (1.20, 1.72)	1.79 (1.66, 2.17)	1.97 (1.89, 2.24)	22.09	<0.001
PA (°)	16.89 ± 2.15^ab^	20.18 ± 1.93	21.30 ± 1.80	24.92	<0.001
CSA (cm^2^)	7.30 ± 1.81^ab^	9.44 ± 1.96^b^	10.70 ± 2.28	13.55	<0.001
MT/HT (cm/m)	0.81^ab^ (0.73, 1.03)	1.08 (0.96, 1.28)	1.16 (1.12, 1.37)	21.77	<0.001
MT/BMI (cm/kg/m^2^)	0.06 ± 0.01^ab^	0.08 ± 0.01	0.09 ± 0.01	15.33	<0.001
CSA/HT (cm^2^/m)	4.38 ± 1.07^ab^	5.61 ± 1.12^b^	6.37 ± 1.27	14.12	<0.001
CSA/BMI (cm^2^/kg/m^2^)	0.34 ± 0.06^ab^	0.42 ± 0.06^b^	0.47 ± 0.09	14.86	<0.001

^a^Compare with the group of non-CTM *P* < 0.05;^ b^Compare with the group of health control *P* < 0.05.

BMI, body mass index; CSA, cross-sectional area; CTM, chronic thyrotoxic myopathy; HC, healthy control; HT, height; MT, muscle thickness; non-CTM, non-chronic thyrotoxic myopathy; PA, pennation; SFT, subcutaneous fat thickness; VL, vastus lateralis; VM, vastus medialis.

**Table 4:** Ultrasound measurements of muscular parameters between the different sex groups of CTM participants.

	Male (*n* = 18)	Female (*n* = 49)	t/Z	*P*
**VL**				
SFT (cm)	0.92 ± 0.40	1.53 ± 0.49	−4.59	<0.001
MT (cm)	1.79 ± 0.44	1.67 ± 0.35	1.16	0.250
PA (°)	16.85 ± 2.74	15.70 ± 2.13	1.80	0.076
MT/HT (cm/m)	1.07 ± 0.26	1.06 ± 0.22	0.93	0.09
MT/BMI (cm/kg/m^2^)	0.08 ± 0.01	0.08 ± 0.01	−0.13	0.898
**VM**				
SFT (cm)	1.09 ± 0.44	1.68 ± 0.48	−4.41	<0.001
MT (cm)	1.44 ± 0.30	1.30 ± 0.33	0.74	0.125
PA (°)	16.89 ± 2.15	15.03 ± 2.65	2.59	0.012
CSA (cm^2^)	7.30 ± 1.81	5.25 ± 1.20	5.26	<0.001
MT/HT (cm/m)	0.86 ± 0.17	0.81 ± 0.18	0.93	0.356
MT/BMI (cm/kg/m^2^)	0.06 (0.05, 0.07)	0.06 (0.05, 0.07)	363.00	0.579
CSA/HT (cm^2^/m)	4.38 ± 1.07	3.34 ± 0.73	4.43	<0.001
CSA/BMI (cm^2^/kg/m^2^)	0.35 (0.29, 0.40)	0.26 (0.22, 0.31)	163.00	<0.001

BMI, body mass index; CSA, cross-sectional area; HC, healthy control; HT, height; MT, muscle thickness; PA, pennation; SFT, subcutaneous fat thickness; VL, vastus lateralis; VM, vastus medialis.

**Figure 4:**
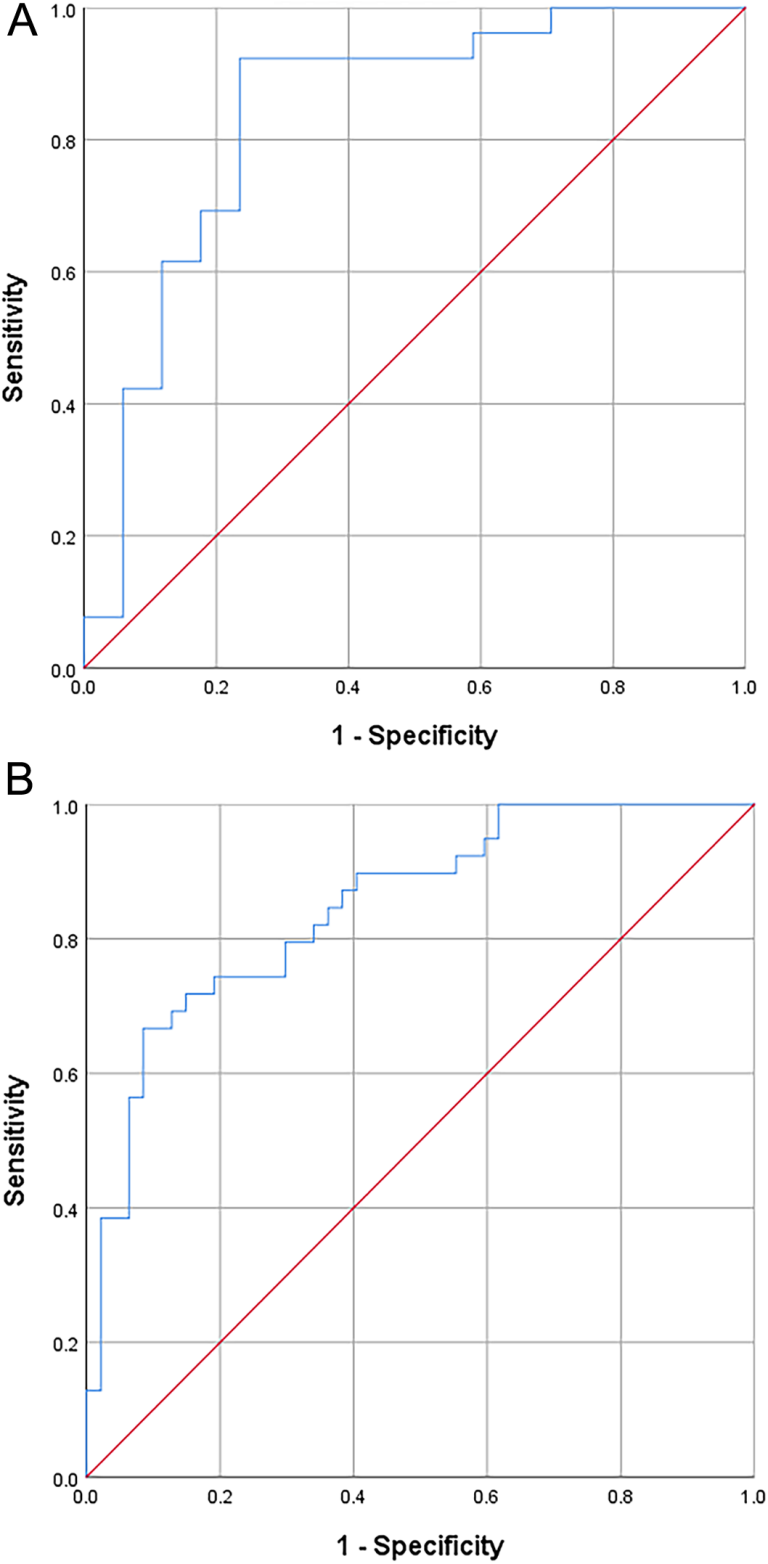
ROC curves of the variables with the maximum sex-specific AUCs for the diagnosis of CTM. A. VM-MT/BMI in males. B. VM-CSA/HT in females.

According to the ROC analysis, the MT/HT and MT/BMI of the VL and the MT/HT, MT/BMI, CSA/HT and CSA/BMI of the VM were significant predictors of CTM. In female participants, the index with the maximum area under the curve (AUC) was VM-CSA/HT, with an AUC of 0.85. The optimal VM-CSA/HT threshold was 4.01, which had 66.70% sensitivity and 89.40% specificity for the diagnosis of CTM (Fig. 4). In contrast, the variable with the highest AUC (0.84) in males was VM-MT/BMI, with 92.30% sensitivity and 76.50% specificity for the diagnosis of CTM (Fig. 4). In addition, VM-MT/HT had 100.00% sensitivity as a predictor of male CTM. The sex-specific ROC analysis, sensitivity, and specificity of all these muscle parameters are presented in Tables 5 and 6.

**Table 5:** The result of diagnostic value of ultrasound measurements of muscular parameters in females.

	AUC	95% CI	*P*	Cutoff	Sensitivity (%)	Specificity (%)	Youden index	PPV (%)	NPV (%)
VL-MT	0.69	0.57–0.80	0.001	1.76	65.70	63.80	0.30	69.51	59.69
VL-MT/HT	0.69	0.57–0.80	0.002	1.13	74.30	61.70	0.36	70.91	65.65
VL-MT/BMI	0.64	0.53–0.76	0.018	0.09	68.60	59.60	0.28	68.08	60.17
VM-MT	0.71	0.60–0.82	<0.001	1.29	82.10	53.20	0.35	68.79	70.29
VM-MT/HT	0.71	0.60–0.82	<0.001	0.89	69.20	68.10	0.37	71.24	59.37
VM-MT/BMI	0.67	0.54–0.77	0.014	0.07	71.30	57.40	0.32	73.22	63.80
VM-CSA	0.85	0.77–0.93	<0.001	6.26	69.20	89.40	0.55	67.77	61.42
VM-CSA/HT	0.85	0.77–0.93	<0.001	4.01	66.70	91.50	0.58	90.79	68.79
VM-CSA/BMI	0.76	0.66–0.86	<0.001	0.27	92.30	53.20	0.46	71.25	84.61

AUC, area under curve; BMI, body mass index; CSA, cross-sectional area; HT, height; MT, muscle thickness; NPV, negative predictive values; PPV, positive predictive values; VL, vastus lateralis; VM, vastus medialis.

**Table 6:** The result of diagnostic value of ultrasound measurements of muscular parameters in males.

	AUC	95% CI	*P*	Cutoff	Sensitivity (%)	Specificity (%)	Youden index	PPV (%)	NPV (%)
VL-MT	0.75	0.60–0.89	0.006	1.90	83.30	58.80	0.42	56.52	84.56
VL-MT/HT	0.71	0.56–0.87	0.018	1.11	76.90	61.10	0.38	55.96	80.45
VL-MT/BMI	0.78	0.64–0.91	0.002	0.09	69.20	77.80	0.47	66.71	79.71
VM-MT	0.81	0.68–0.95	0.001	1.41	100.00	58.80	0.59	60.94	100.00
VM-MT/HT	0.82	0.69–0.95	<0.001	0.83	100.00	58.80	0.59	60.94	100.00
VM-MT/BMI	0.84	0.71–0.97	<0.001	0.07	92.30	76.50	0.68	71.63	93.92
VM-CSA	0.79	0.65–0.93	0.001	7.96	76.90	77.80	0.56	69.01	83.97
VM-CSA/HT	0.78	0.64–0.92	0.002	4.95	73.10	77.80	0.52	67.92	81.82
VM-CSA/BMI	0.79	0.65–0.92	0.001	0.42	53.80	94.40	0.48	86.06	76.07

AUC, area under curve; BMI, body mass index; CSA, cross-sectional area; HT, height; MT, muscle thickness; NPV, negative predictive values; PPV, positive predictive values; VL, vastus lateralis; VM, vastus medialis.

The US-derived parameters VM-MT, VM-MT/BMI, VM-MT/HT, VM-CSA, and VM-CSA/HT were negatively correlated with FT4 in both groups (*P* < 0.05), while VL-MT, VL-MT/HT, VL-MT/BMI, and VM-CSA/BMI were negatively correlated with FT4 in males (*P* < 0.05) but not in females (*P* > 0.05). VM-MT, VM-MT/HT, VM-CSA, and VM-CSA/HT were negatively correlated with TRAb in both groups (*P* < 0.05). Weight and BMI were positively correlated with MT, MT/HT, CSA, and CSA/HT in both groups (*P* < 0.05). Details of the correlation analysis for the MT, CSA and corrected parameters are shown in Tables 7 and 8.

**Table 7:** Correlation analysis results between US correct parameters and the level of thyroid hormone, FT4, TRAb, weight, and BMI in females.

	Duration		FT4		TRAb		Weight		BMI	
	*r*	*P*	*r*	*P*	*r*	*P*	*r*	*P*	*r*	*P*
VL-MT	−0.14	0.249	−0.18	0.116	−0.13	0.244	0.51	<0.001	0.50	<0.001
VL-MT/HT	−0.11	0.377	−0.18	0.107	−0.13	0.253	0.46	<0.001	0.53	<0.001
VL-MT/BMI	−0.03	0.798	−0.07	0.528	−0.08	0.497	−0.10	0.414	−0.20	0.075
VM-MT	−0.28	0.021	−0.35	0.001	−0.36	0.001	0.38	<0.001	0.30	0.006
VM-MT/HT	−0.22	0.070	−0.30	0.004	−0.32	0.004	0.30	0.005	0.31	0.004
VM-MT/BMI	−0.17	0.161	−0.24	0.028	−0.20	0.075	−0.23	0.037	−0.36	0.001
VM-CSA	−0.13	0.306	−0.31	0.004	−0.30	0.007	0.45	<0.001	0.37	<0.001
VM-CSA/HT	−0.08	0.550	−0.29	0.007	−0.27	0.016	0.38	<0.001	0.37	<0.001
VM-CSA/BMI	−0.01	0.957	−0.15	0.157	−0.161	0.151	−0.14	0.197	−0.28	0.010

BMI, body mass index; CSA, cross-sectional area; FT4, free thyroxine; HT, height; MT, muscle thickness; RAb, thyroid-stimulating hormone receptor antibody; VL, vastus lateralis; VM, vastus medialis.

**Table 8:** Correlation analysis results between US correct parameters and the level of thyroid hormone, FT4, TRAb, weight, and BMI in males.

	Duration		FT4		TRAb		Weight		BMI	
	*r*	*P*	*r*	*P*	*r*	*P*	*r*	*P*	*r*	*P*
VL-MT	−0.24	0.165	−0.36	0.018	−0.25	0.108	0.65	<0.001	0.70	<0.001
VL-MT/HT	−0.24	0.168	−0.33	0.032	−0.24	0.110	0.61	<0.001	0.71	<0.001
VL-MT/BMI	−0.21	0.221	−0.36	0.016	−0.16	0.305	0.19	0.230	0.17	0.278
VM-MT	−0.31	0.069	−0.44	0.003	−0.41	0.008	0.48	0.001	0.58	<0.001
VM-MT/HT	−0.32	0.058	−0.44	0.003	−0.39	0.011	0.39	0.010	0.55	<0.001
VM-MT/BMI	−0.31	0.067	−0.47	0.002	−0.23	0.149	−0.09	0.559	−0.08	0.607
VM-CSA	−0.30	0.075	−0.32	0.032	−0.47	0.002	0.67	<0.001	0.66	<0.001
VM-CSA/HT	−0.31	0.065	−0.33	0.028	−0.46	0.002	0.64	<0.001	0.68	<0.001
VM-CSA/BMI	−0.30	0.074	−0.35	0.021	−0.38	0.011	0.22	0.147	0.15	0.343

BMI, body mass index; CSA, cross-sectional area; FT4, free thyroxine; HT, height; MT, muscle thickness; RAb, thyroid-stimulating hormone receptor antibody; VL, vastus lateralis; VM, vastus medialis.

## Discussion

The main finding of this study was that muscular US is a highly reproducible method of quantifying the local MT, PA, and CSA of proximal muscles in hyperthyroid patients with or without CTM. This is the first study to investigate the US characteristics of muscles in patients with CTM. Moreover, the current results demonstrated a significant correlation between US parameters and thyroid hormone levels, indicating that the MT, PA, and CSA are associated with a thyroid hormone increase in patients with hyperthyroidism. In addition, VM-CSA/HT and VM-MT/BMI were found to be the best muscle US parameters for predicting CTM in female and male hyperthyroid patients, respectively.

The weight, BMI, WC, GC, and MTC of the CTM group were lower than those of the non-CTM and HC groups. In contrast, the thyroid hormone levels in the CTM group were higher than those in the non-CTM group. Weight was negatively correlated with thyroid hormone levels, meaning that the higher a hyperthyroid patient’s FT3 and FT4 levels were, the lower that patient’s weight tended to be. This result indicates that thyroid hormones decrease weight in a dose-dependent manner. Soriguer *et al.* (35) found that lower levels of thyroid hormones were associated with a greater BMI in humans. Marzullo *et al.* (36) demonstrated a positive relationship between thyroid hormone levels and resting energy expenditure during weight loss. The functions of the thyroid include the regulation of thermogenesis and body weight (37). Thyroid hormones increase the basal metabolic rate by increasing the gene expression of Na^+^–K^+^ ATPase in different tissues (38). In addition, an excess of thyroid hormones shifts the nitrogen balance in the negative direction. In general, patients with a lower weight or BMI tend to have significantly lower skeletal muscle mass (39). This observation may illustrate the pathogenesis and mechanism of CTM and support the association between weight and thyroid hormone levels in CTM. In brief, the present study is the first to explore the relationship of weight and BMI with thyroid hormone levels in CTM.

Ramsay *et al.* (40) reported that the proximal muscles of the shoulders, hand, and pelvic girdle are most commonly affected in CTM. Matsuki *et al.* (41) reported that upper arm circumference was a good index of body muscle mass based on a study of 401 untreated patients with thyrotoxicosis and the same number of control subjects; their findings suggest that upper arm circumference and body muscle mass are lower in patients with thyrotoxicosis than in controls. These reports from prior investigations are consistent with the results of our research. Our study provided evidence of several novel characteristics of CTM: reduced WC, GC, and MTC, reflecting proximal muscle weakness or atrophy.

Previous studies have found that US images have strong agreement with traditional techniques for measuring muscle mass. Swanson *et al.* (42) demonstrated US as a valid and reliable alternative to MRI in measuring the CSA of foot muscles. P. Casey *et al.* (43) suggested that US could detect alterations in muscle and assess muscle quality and quantity in acute and chronic clinical conditions. P. Ritsche *et al.* (13) provided evidence for the agreement and reliability of lower limb muscle architecture measurements using a portable US device by assessing healthy participants. However, no articles have reported the use of muscular US to predict clinical and functional outcomes in CTM. To fill this gap, we measured the architectural parameters of skeletal muscle by US and developed new sonography-based diagnostic criteria for CTM. Alayli *et al.* (44) reported that the muscle CSA corrected for BMI (CSA/BMI) had a close association with physical function tests and quadriceps muscle strength. Kim *et al.* (45) utilized the thickness of the psoas muscle divided by height (MT/HT) to evaluate sarcopenia. Wang *et al.* (14) reported that the corrected limb US measurement indexes, such as MT/BMI and MT/SFT, are reliable and accurate measures for assessing skeletal muscle. Among the indicators analyzed in this study, the US measurements relevant to CTM diagnosis included MT, PA, CSA, MT/HT, MT/BMI, CSA, CSA/HT, and CSA/BMI.

Another interesting finding of our study is the fact that thyroid hormone levels were negatively correlated with some muscular US measurements. This finding is consistent with that of Somay *et al.*, whose results showed that almost 2/3 of patients had clinical weakness in at least one muscle group that correlated with free thyroxine concentrations (46). Lovejoy *et al.* (47) indicated that high levels of T3 in serum lessened both muscle and fat mass. Martin *et al.* (48) found that participants who took 100 mg of T3 daily for 2 weeks had decreased skeletal muscle mass, which resulted in impaired exercise tolerance. The architecture and pathology of muscle tissue in CTM directly determine the functional impairment of skeletal muscles. Previous articles have suggested that coupled oxidative phosphorylation and uncoupled respiration may play an important role in the impairment of mitochondrial energy metabolism in skeletal muscles (49, 50). Excess thyroid hormone levels accelerate protein catabolism and reduce oxidative capacity, resulting in skeletal muscle dysfunction (48). Excess thyroid hormone levels disrupt the composition of muscle tissue, increasing the frequency of type 2 fibers, as observed microscopically in muscle biopsy samples (51). In addition, reduced membrane excitability and diminished power of contraction due to an increase in the rate of relaxation lead to muscle weakness in hyperthyroidism (52). These findings from previous studies suggest that excess thyroid hormone could alter the function, architecture, and pathology of skeletal muscle and then promote the occurrence of CTM. However, Kung *et al.* (53) proposed that the extent and severity of myopathy in thyrotoxicosis are related to the duration of hyperthyroidism, not the severity of biochemical anomalies. We also found that CTM patients had a longer duration of hyperthyroidism than non-CTM hyperthyroid patients, but we did not find a significant correlation between the duration of hyperthyroidism and any of the US indexes in this study. Consequently, based on these previously proposed theories and our current findings, skeletal muscle function in CTM can be indirectly reflected by the feasible and broadly applicable technique of US. For this reason, we plan to use US in our future work to conveniently evaluate muscle architecture and reflect skeletal muscle function in CTM.

Additionally, we found that TRAb levels in the CTM group were higher than those in the non-CTM and HC groups. The relationship between TRAb and some muscular US measurements became negative. Aside from the deterioration of muscle fibers, CTM also exhibits pathological changes that involve lymphocyte infiltration, suggesting a possible involvement of autoantibodies in the development of CTM (53). Elevated TRAb levels in individuals with CTM may excessively trigger thyroid follicular cells, which produce and release more thyroid hormones, indirectly contributing to the development of CTM (54).

Our results show that, as estimated by US, the VM and VL of CTM patients have less muscle mass than those of non-CTM patients and HCs. The patients were divided into two groups (male and female) to eliminate the influence of sex on the skeletal muscle. This is the first study to investigate muscle mass by muscular US in patients with CTM. Our study indicated that more muscle MS indexes were significantly associated with CTM in females than in males, which means that female skeletal muscle seems more affected by hyperthyroidism. We speculate that this is related to the difference in muscle reserve caused by the different sex hormone profiles of men and women, as well as a greater frequency of moderate to intense physical activity by males than by females in the long term. Harman *et al.* (55) stated that sex hormone changes may influence lean body mass and strength. H. Rakov *et al.* (56) observed decreased muscle strength and motor coordination specifically in male hyperthyroid mice and not in females. Kung *et al.* (53) stated that symptomatic myopathy is more common among men than women. Our experiment challenges existing theories by suggesting that thyrotoxicosis is, in fact, more common in women than men.

Moreover, we compared the performance of the MT, PA, and CSA as well as the corrected indexes for assessing CTM. Our results indicated that the presence or absence of CTM should be a greater concern in the clinical diagnosis of female thyrotoxic patients. The muscle with the greatest AUC for predicting CTM in males and females was the VM. The cutoff values of VM-MT/BMI in males and VM-CSA/HT in females were 0.07 and 4.01, respectively. The results of the ROC curve analysis showed that VM-MT/BMI in males and VM-CSA/HT in females were excellent indicators of CTM. The VM had a larger AUC value than the VL. Thus, VM may be the better choice for US-based prediction of CTM in hyperthyroidism.

This is the first diagnostic effectiveness analysis of the architecture of susceptible muscles in CTM based on US measurements. Some limitations of our study should be noted. First, the sample size of our research is small, and all subjects were from a single center. Second, this study examined only a subset of the proximal muscles. Examining other pelvic muscles and scapular girdle muscles may lead to different findings. Third, this study omitted some US variables, such as muscle fascicle length and echogenicity.

In summary, we draw the following conclusions. First, the reduced weight, BMI, and body circumferences in CTM compared with non-CTM hyperthyroid patients are related to increased thyroid hormone levels. Second, the validity and feasibility of US for assessing the characteristics of the VL and VM muscles in CTM are demonstrated. Some muscular US parameters were lower in CTM than in non-CTM hyperthyroid patients in our study, and some US parameters of the VM were lower in female than in male CTM patients. Muscle US is a promising method for assessing CTM to reflect skeletal muscle mass. Third, according to the results of this study, hyperthyroid patients with a VM-CSA/HT less than 4.01 in females or a VM-MT/BMI less than 0.07 in males can be considered to have CTM. Larger samples from multiple regions are needed to select appropriate diagnostic cutoff values for CTM. Our hope is that US measurements will provide practical benefits in CTM diagnosis.

## Declarations of interest

The authors declare that they have no conflicts of interest.

## Funding

This work was supported by the National Natural Science Foundation of China (Grant No. 82260159, No. 81860146, and No. 81660138) and the Ministry of Science and Technology (grant no. 2016YFC0901205 and no. 2016TFC0901200). The funders had no role in the design of the study; in the collection, analyses, or interpretation of data; in the writing of the manuscript; or in the decision to publish the results.

## Author contribution statement

S.-E.F., X.-H.L., J.X., X.-L. C, Y.-F.Q., H.-Y. Y, L.-L.H., Y.-Q.K., Z.-J.L. contributed to the diagnosis and treatments of hyperthyroidism and CTM; R.-M.W., S.-X.L., C.-C.Q., Z.-P.T., Y.M. contributed to collection of measurements of parameters in US; J.P. performed electromyography; S.-E.F. and R.-M.W. carried out data analysis and data interpretation; S.-E.F. drafted the manuscript. All authors were involved in the design of the study, critical revision of the manuscript, and approval of the final manuscript.
